# Gas-Sensing Properties of Co_9_S_8_ Films Toward Formaldehyde, Ethanol, and Hydrogen Sulfide

**DOI:** 10.3390/ma17235743

**Published:** 2024-11-24

**Authors:** Myeong Gyu Kim, Yun-Hyuk Choi

**Affiliations:** 1 Department of Advanced Materials and Chemical Engineering, Graduate School, Daegu Catholic University, Gyeongsan 38430, Gyeongbuk, Republic of Korea; 2 Department of Energy Materials, Daegu Catholic University, Gyeongsan 38430, Gyeongbuk, Republic of Korea; 3 Department of Battery Engineering, Daegu Catholic University, Gyeongsan 38430, Gyeongbuk, Republic of Korea; 4 Department of Materials Science and Engineering, Daegu Catholic University, Gyeongsan 38430, Gyeongbuk, Republic of Korea

**Keywords:** Co_9_S_8_, gas sensor, formaldehyde, ethanol, hydrogen sulfide

## Abstract

The chemiresistive gas-sensing properties of pristine Co_9_S_8_ film are little known despite its potential as a promising gas sensor material due to its intrinsic characteristics. In this study, a pristine polycrystalline Co_9_S_8_ film (approximately 440 nm in thickness) is fabricated by depositing a Co_3_O_4_ film followed by sulfidation to investigate its gas-sensing properties. The prepared Co_9_S_8_ film sensor is found to exhibit high responsiveness towards formaldehyde (HCHO), ethanol (C_2_H_5_OH), and hydrogen sulfide (H_2_S) at operating temperatures of 300 °C and 400 °C, with strong concentration dependence. On the other hand, the sensor shows very low or no responsiveness towards hydrogen (H_2_), acetone (CH_3_COCH_3_), and nitrogen dioxide (NO_2_). These results enhance our understanding of the intrinsic gas-sensing properties of Co_9_S_8_, aiding in the design and fabrication of high-performance chemiresistive gas sensors based on Co_9_S_8_.

## 1. Introduction

Semiconductor-type chemiresistive gas sensors have been widely investigated and used owing to their simplicity, cost-effectiveness in processing and structure, and high sensitivity for detecting hazardous volatile organic compounds (VOCs) and bio-signals [1,2,3,4,5,6]. Various metal oxides, such as n-type SnO_2_, In_2_O_3_, TiO_2_, ZnO, and WO_3_, as well as p-type CuO, Cu_2_O, Cr_2_O_3_, NiO, Co_3_O_4_, Fe_2_O_3_, and Fe_3_O_4_, have been selected as the main sensing materials for such gas sensors [7,8,9,10,11,12,13,14]. To improve performance, these sensing materials have been further engineered into forms such as n-n-, p-p-, and n-p-type heterostructures [15,16], nanostructures [17,18,19,20], functionalized surfaces [21,22], and non-stoichiometric compositions [23,24,25]. Another significant area of study is the exploration of new phases and compositions (using multi-components) with excellent sensor characteristics [26,27]. In this respect, metal sulfides have been emerging as a very promising group of gas sensor materials that can potentially replace oxides. Metal sulfides are different from metal oxides in terms of chemical reactivity due to the higher average polarizability (2.90 × 10^−24^ cm^3^) of sulfur compared to oxygen (0.802 × 10^−24^ cm^3^) [28]. This results in the lower electronegativity of sulfur (2.5) compared to oxygen (3.5), indicating that the metal–sulfur bond is more covalent than the metal–oxygen bond [28]. In addition, metal sulfides possess suitable electronic band gaps, favorable band positions, exposed active sites, high photosensitivity, large specific capacities, low redox potential, low melting points, nanocrystalline morphology, and a longer lifespan compared to metal oxides [28]. They are also abundant and cheap since they usually exist in nature as minerals [29]. Recently, various metal sulfides with specific compositions, such as PbS, Cu_x_S, CdS, SnS_2_, ZnS, WS_2_, and MoS_2_, have been studied for application in gas sensors [30]. However, studies on such metal sulfide-based gas sensors are still limited to only a few compositions. Therefore, the gas sensor characteristics for a wider range of compositions and phases in metal sulfides should be further investigated in the future.

Binary cobalt sulfides exist in the form of various crystalline phases in a wide range of stoichiometries, including CoS, Co_2_S, CoS_2_, Co_2_S_3_, Co_3_S_4_, and Co_9_S_8_, and in nonstoichiometric Co_1−x_S [31,32,33,34]. Among them, although studies of Co_9_S_8_ are relatively rare, we were inspired by its use as a superior catalyst and investigated it as a promising gas sensor material in this study. Co_9_S_8_ has high electrical conductivity (0.29 × 10^3^ S cm^−1^ at room temperature) and multiple active metal sites, which are expected to exhibit excellent adsorption and desorption reactions to gas species [35]. Co_9_S_8_ has a face-centered cubic (fcc) structure, where the Co tetrahedron and octahedron are coordinated to four S ions and six S ions, respectively [31,32,36]. They coexist with the Co octahedron located at the center and the edge centers of the cubic cell, while every eight closely packed Co tetrahedrons are located at the vertices and face centers. Interestingly, it exhibits unique size-dependent band gap characteristics, where the band gap gradually increases from 0.97 eV [37] to 1.01 eV [38], 1.23 eV [39], 1.42 eV [40], 1.61 eV [41], and 1.78 eV [42] as the particle size decreases from bulk to nanodots. Co_9_S_8_ has been applied in various fields, including catalysis [31,43], electrocatalysis [32,34,36,44,45,46,47,48], photocatalysis [37,38,39,40,41,42], solar cells [31,33], supercapacitors [35,49,50], and batteries [48,51]. Surprisingly, however, little is known about its application as a gas sensor and its gas-sensing properties. Electrochemical sensors using Co_9_S_8_ for the detection of luteolin, metronidazole, hydrogen peroxide, and glucose have been reported [52,53,54]. Photoelectrochemical sensors using Co_9_S_8_ for the detection of chlorpyrifos and sulfamethazine have recently been reported in the form of Z-scheme heterostructures [55,56]. To the best of our knowledge, only three studies on semiconductor-type chemiresistive gas sensors using Co_9_S_8_ have been conducted [57,58,59].

In this study, the chemiresistive gas-sensing properties of pristine Co_9_S_8_ film are investigated for formaldehyde (HCHO), ethanol (C_2_H_5_OH), hydrogen sulfide (H_2_S), hydrogen (H_2_), acetone (CH_3_COCH_3_), and nitrogen dioxide (NO_2_) at various operating temperatures and gas concentrations. In particular, promisingly high responsiveness to HCHO, C_2_H_5_OH, and H_2_S with high selectivity is found. Given the relatively few studies on the gas-sensing characteristics of Co_9_S_8_, this work systematically investigates its gas-sensing properties. Notably, it demonstrates that Co_9_S_8_ gas sensors exhibit high responsiveness to HCHO and H₂S gasses, which have not been observed in the limited prior reports. From this perspective, these findings underscore the need for further exploration and highlight the potential of Co_9_S_8_ in gas-sensing applications.

Furthermore, as is well known, wet chemical deposition methods offer relatively low-cost, high-throughput film fabrication, whereas vacuum deposition techniques, such as CVD and magnetron sputtering, are expensive and sensitive to contaminants, requiring high-temperature and low-pressure conditions. However, vacuum deposition methods provide significant advantages in terms of high uniformity and large-scale film fabrication, making them widely used in the semiconductor and display industries. In this work, we developed a thermal metal–organic decomposition (MOD) process to prepare single-phase Co_9_S_8_ films for gas sensor applications. This process was designed to enhance practical usability in industrial applications.

## 2. Experimental Section

### 2.1. Stepwise Deposition from Co_3_O_4_ to Co_9_S_8_ Films

The preparation of Co_9_S_8_ films involves a two-step process: the thermal metal–organic deposition (MOD) of Co_3_O_4_ films, followed by sulfidation. To initiate this process, Co_3_O_4_ films were deposited on Si/SiO_2_ substrates (2 μm-thick oxide) using the MOD process, following a previously reported procedure [60]. As illustrated in Figure S1a (Supplementary Materials), the thermal MOD process was conducted using a horizontal cold-wall quartz tube furnace measuring 32 mm in diameter and 600 mm in length, equipped with gas flow controls. The furnace had a length of 400 mm, with both ends of the quartz tube extending outside the furnace by 100 mm each. For the deposition of Co_3_O_4_ films, cobalt(II) acetate tetrahydrate powder (Daejung (Siheung-si, Republic of Korea), ≥98% purity) was utilized as a precursor. Various amounts of the precursor were tested for film deposition, including 10 mg, 20 mg, 30 mg, and 40 mg. The precursor was placed inside an alumina boat measuring 50 mm in length, 10 mm in width, and 9 mm in height. This boat was positioned at the center of the tube. A Si/SiO_2_ substrate, measuring 2 cm in length and 2 cm in width, covered the alumina boat. The deposition side of the substrate was positioned facing down, looking at the precursor. After an initial Ar purge for 30 min, the furnace was heated to 500 °C at a ramp rate of 20 °C/min under a 30 cc/min Ar flow at 1 atm. After holding the furnace at 500 °C for 10 min, it was allowed to cool naturally to room temperature.

As depicted in Figure S1b, in the subsequent step, the Co_3_O_4_-coated Si/SiO_2_ substrate was positioned at the center of the tube furnace, with the Co_3_O_4_ coating side facing up. An alumina boat containing 100 mg of elemental sulfur powder (Alfa Aesar (Haverhill, MA, USA), 99.5% purity) was positioned at the end of an electric furnace in the direction of the Ar upstream, followed by an Ar purge for the initial 30 min. Subsequently, the furnace was heated to 600 °C at a ramp rate of 20 °C/min under a 50 cc/min Ar flow at 1 atm. After holding the furnace at 600 °C for 10 min, it was allowed to cool naturally to room temperature. This process allowed for the transformation of Co_3_O_4_ into Co_9_S_8_ within the film.

### 2.2. Structural Characterization

The phase of the films was investigated using the X-ray diffraction (XRD) θ–2θ method with a Cu K_α_ source (λ = 1.5406 Å) on an X’pert Pro instrument from PANalytical (Malvern, UK). The film morphology and composition were observed using the field-emission scanning electron microscopy (FE-SEM) and energy-dispersive spectroscopy (EDS) techniques, respectively, employing a Hitachi S-4800 instrument (Hitachi High-Tech Corporation, Tokyo, Japan). The crystal structure was further investigated using high-resolution transmission electron microscopy (HRTEM) with an FEI Titan G2 60-300 S/TEM (FEI Company, Thermo Fisher Scientific, Waltham, MA, USA) instrument operated at an accelerating voltage of 200 kV. The instrument was equipped with a spherical-aberration (Cs) probe corrector and ChemiSTEM technology. The surface chemical state was examined using X-ray photoelectron spectroscopy (XPS) with monochromatized Al K_α_ radiation, conducted on a Thermo Scientific instrument (Waltham, MA, USA). Energy calibration was accomplished by setting the hydrocarbon C 1s line at 284.80 eV. The thermal decomposition behavior of the deposit on the substrate was investigated using thermogravimetric analysis (TGA) and derivative thermogravimetry (DTG) on a Discovery SDT 650 instrument from TA Instruments (New Castle, DE, USA). The analysis was conducted in the temperature range of 25 to 600 °C at a scan rate of 10 °C/min, utilizing Tzero aluminum pans in both air and N_2_ atmospheres.

### 2.3. Fabrication and Measurement of Gas Sensor

As shown in Figure S1c, for the fabrication of the gas sensor, a pair of comb-like Pt electrodes were deposited onto the surface of the film formed on the Si/SiO_2_ substrate via sputtering through a mask. The gap between the Pt electrodes was 0.2 mm, and their width was 8 mm. Afterwards, Au wires were affixed to the electrodes using silver and alumina pastes, followed by firing at 600 °C for 1 h without any alteration in morphology or phase. The sensor was placed in a quartz tube located inside an electrical tube furnace with a gas inlet/outlet system (Figure S1d). The gas-sensing properties of the sensor were investigated by measuring the changes in electrical resistance towards various gasses, including 50–1000 ppm acetone (CH_3_COCH_3_), 50–1000 ppm hydrogen (H_2_), 10–500 ppm formaldehyde (HCHO), 10–1000 ppm ethanol (C_2_H_5_OH), 1–10 ppm nitrogen dioxide (NO_2_), and 1–10 ppm hydrogen sulfide (H_2_S), balanced with air, and pure air at operating temperatures ranging from 200 °C to 600 °C. The magnitude of the gas response (S) was defined as the ratio (R_a_/R_g_) of the resistance in air (R_a_) to that in the sample gas (R_g_). The response time (t_90%_) was defined as the time required for the sensor to reach 90% of the final signal. All gas response and response time values are reported as the mean and standard deviation of measurements obtained from each of the three sensors fabricated under identical conditions.

## 3. Results and Discussion

### 3.1. Structural Characteristics

To prepare the Co_9_S_8_ film, Co_3_O_4_ films were initially deposited on Si/SiO_2_ substrates through the MOD process using various amounts of cobalt(II) acetate tetrahydrate as the precursor, specifically, 10 mg, 20 mg, 30 mg, and 40 mg. Interestingly, as shown in Figure S2, the morphology of the deposits varies with the amount of the precursor. The deposits made using 10 mg and 20 mg of the precursor are in the form of a film, with particle sizes of 300–500 nm and film thicknesses of 250–270 nm (Figure S2a,b). In particular, for the 10 mg precursor, the secondary particles consist of smaller primary particles, while for the 20 mg precursor, it is observed that sintering occurs with necking among the particles. For the 30 mg precursor, rough particles with a diameter of 550–750 nm are thinly formed on the substrate, reaching a height of 897 nm (Figure S2c). For the 40 mg precursor, large secondary particles with a diameter of 2–3 μm are very sparsely formed on the substrate, which are composed of small primary particles ranging from 130 to 245 nm in diameter (Figure S2d). In this work, we selected the Co_3_O_4_ film prepared using the 10 mg cobalt(II) acetate tetrahydrate precursor as a pre-stage film to ultimately fabricate the Co_9_S_8_ film, due to its uniform and dense film morphology.

**Figure 1:**
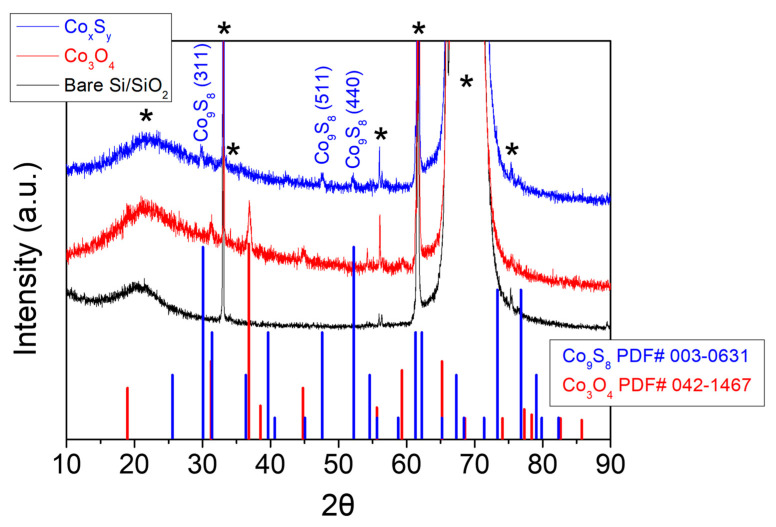
The XRD patterns acquired for the prepared Co_3_O_4_ and Co_9_S_8_ films, as well as a bare Si/SiO_2_ substrate. Peaks corresponding to the respective PDF numbers for Co_3_O_4_ and Co_9_S_8_ are shown together below the graph. In the graph, the asterisks are denoted as the peaks arising from the Si/SiO_2_ substrate.

**Figure 2:**
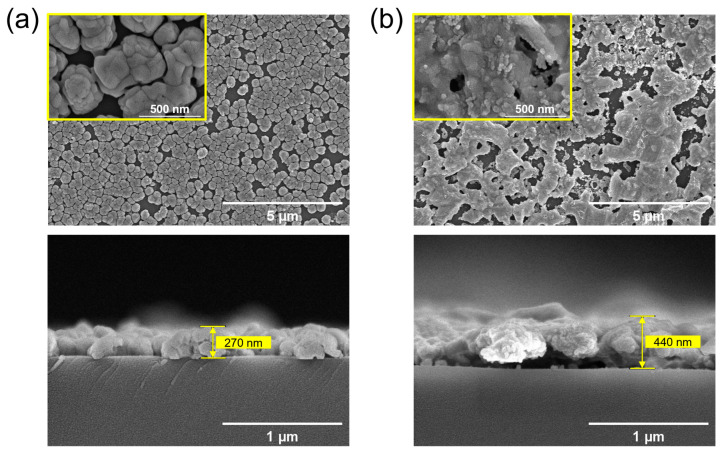
(Top) Out-of-plane and (bottom) in-plane FE-SEM images of the prepared (**a**) Co_3_O_4_ and (**b**) Co_9_S_8_ films formed on Si/SiO_2_ substrates. The insets in the top images are high-magnification images.

The Co_3_O_4_ film deposited on the substrate was subsequently subjected to sulfidation through the vapor transport of elemental sulfur. In Figure 1, the characteristic XRD patterns of the prepared Co_3_O_4_ and Co_9_S_8_ films are shown, despite strong interference peaks originating from the Si/SiO_2_ substrate. Both the Co_3_O_4_ and Co_9_S_8_ films reveal their well-defined polycrystalline structures. Figure 2a,b represent the out-of-plane and in-plane FE-SEM images of the Co_3_O_4_ and Co_9_S_8_ films, formed on Si/SiO_2_ substrates. The Co_3_O_4_ film prepared in the pre-stage exhibits the characteristic microstructure of secondary particles (300–500 nm in diameter) composed of smaller primary particles, with a film thickness of 270 nm as mentioned above (Figure 2a). After sulfidation, a sintered and collapsed morphology among the particles is observed in the formed Co_9_S_8_ film, which expands to a few micrometers in size and 440 nm in film thickness (Figure 2b). The elemental composition of the Co_9_S_8_ film, confirmed by EDS, is shown in Figure S3, alongside Si and O arising from the substrate. These results clearly indicate that the sulfidation process through vapor transport leads to an oxygen–sulfur exchange reaction, transforming Co_3_O_4_ into Co_9_S_8_, as depicted in reactions (1) and (2) [61,62].

3Co_3_O_4_ (s) + 8S (g) → Co_9_S_8_ (s) + 6O_2_ (g) (1)

3Co_3_O_4_ (s) + 8SO_2_ (g) → Co_9_S_8_ (s) + 14O_2_ (g) (2)

**Figure 3:**
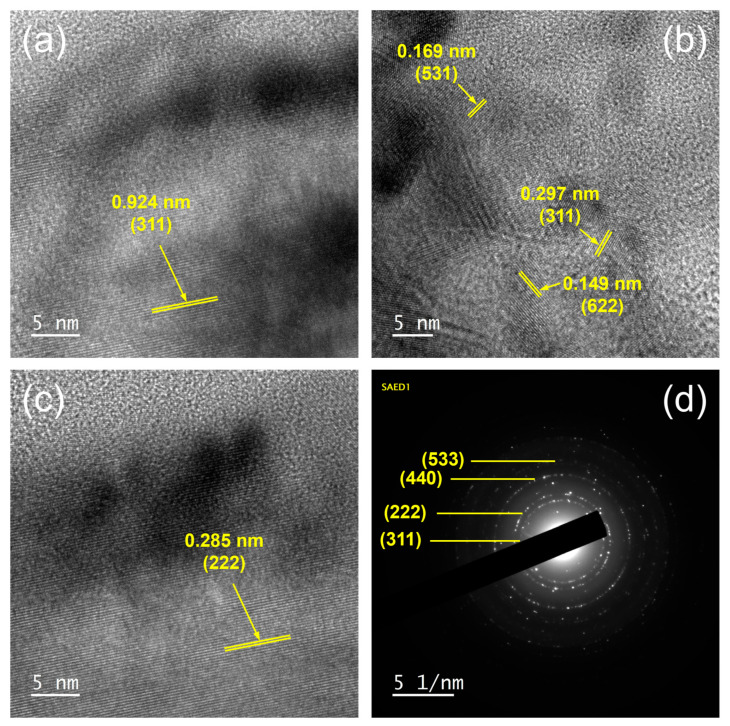
(**a**–**c**) High-resolution TEM lattice fringe images acquired at various positions, and (**d**) SAED pattern of Co_9_S_8_.

The polycrystalline structure of the Co_9_S_8_ film was further identified by HRTEM. As shown in Figure 3a–c, various lattice fringes of Co_9_S_8_ are observed, along with additional lattice planes such as (222), (531), and (622), which are difficult to confirm due to their low intensities or being buried in the substrate peaks in the XRD analysis. Several rings presented in the related SAED pattern are indexed to the (311), (222), (440), and (533) planes of Co_9_S_8_ (Figure 3d).

**Figure 4:**
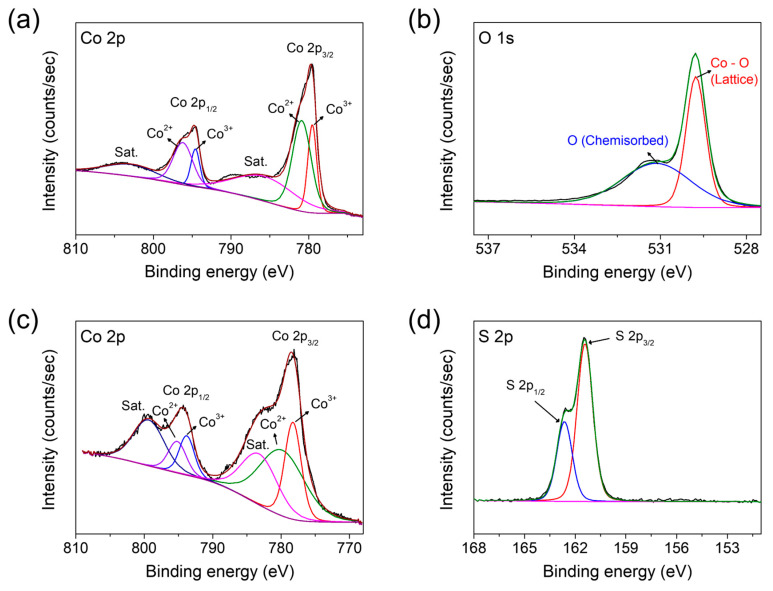
The core-level XPS spectra of (**a**) Co 2p and (**b**) O 1s acquired for the Co_3_O_4_ film, and (**c**) Co 2p and (**d**) S 2p acquired for the Co_9_S_8_ film.

The surface chemical states of the prepared Co_3_O_4_ and Co_9_S_8_ films were investigated using XPS. For the Co_3_O_4_ film, the core-level spectrum of Co 2p reveals two spin–orbit peaks (2p_1/2_ and 2p_3/2_) and two satellite peaks (Figure 4a). The 2p_1/2_ peak is deconvoluted into two sub-peaks around 794.58 eV and 796.28 eV, indicating the oxidation states of Co^3+^ and Co^2+^, respectively. Additionally, the oxidation states of Co^3+^ and Co^2+^ are confirmed by the 2p_3/2_ peak, which is deconvoluted into two sub-peaks around 779.58 eV and 780.88 eV, respectively. The shake-up satellite peaks are detected because the outgoing electron interacts with a valence electron and excites it (shakes it up) to a higher energy level. The presence of two satellite peaks in the Co 2p spectrum indicates that the cobalt is present in a 2+ oxidized state [63]. The O 1s spectrum is deconvoluted into two components: the lattice oxygen at 529.78 eV and the surface-chemisorbed oxygen at 531.18 eV (Figure 4b). These results are in good agreement with the typical chemical states of Co_3_O_4_ [64]. For the Co_9_S_8_ film, the Co 2p spectrum presents two spin–orbit peaks and two satellite peaks, similar to those observed in the Co_3_O_4_ film (Figure 4c). The 2p_1/2_ and 2p_3/2_ peaks are deconvoluted into two sub-peaks each: peaks at 793.80 eV and 778.30 eV arising from Co^3+^ ions, and peaks at 795.30 eV and 780.30 eV arising from Co^2+^ ions within Co_9_S_8_. In Figure 4d, the S 2p spectrum is deconvoluted into 2p_1/2_ and 2p_3/2_ peaks at 162.68 eV and 161.38 eV, respectively. These results are indeed consistent with the reported binding energies of Co_9_S_8_ [44,52,57,58].

**Figure 5:**
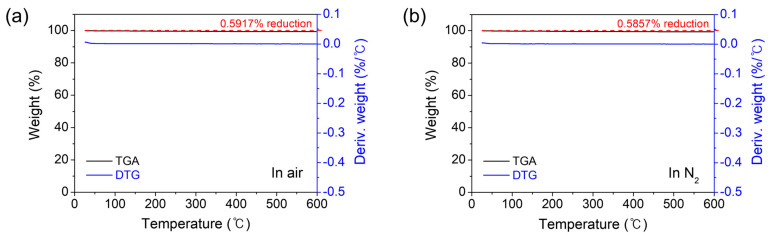
The TGA and DTG profiles obtained for the Co_9_S_8_ film deposited on a Si/SiO_2_ substrate, with the temperature increasing from 25 °C to 600 °C in (**a**) air and (**b**) inert N_2_ atmospheres.

To ensure the effectiveness of the prepared Co_9_S_8_ film for gas-sensing applications, its phase stability should be verified at high temperatures in an air atmosphere. To achieve this, TGA and DTG profiles were obtained for the Co_9_S_8_ film deposited on a Si/SiO_2_ substrate, with the temperature increasing from 25 °C to 600 °C in both air and inert N_2_ atmospheres. As shown in Figure 5a,b, no changes in the TGA and DTG profiles are observed within the measured temperature range, despite negligible weight losses of only 0.5917% in air and 0.5857% in N_2_ in the TGA profiles. This indicates that the Co_9_S_8_ film sensor can operate durably from room temperature to high temperatures of up to 600 °C in ambient air.

### 3.2. Gas-Sensing Properties

**Figure 6:**
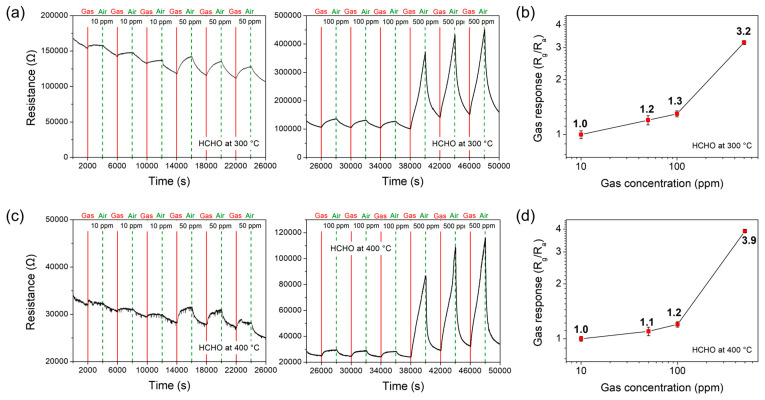
The response transients of the Co_9_S_8_ film sensor which are acquired at various concentrations of 10 ppm, 50 ppm, 100 ppm, and 500 ppm at operating temperatures of (**a**) 300 °C and (**c**) 400 °C towards formaldehyde (HCHO) gas. The gas response values of the sensor are shown as a function of gas concentration on a log–log scale, measured at (**b**) 300 °C and (**d**) 400 °C.

First of all, the gas-sensing properties of the fabricated Co_9_S_8_ film sensor were investigated for formaldehyde (HCHO) balanced with air at operating temperatures ranging from 200 °C to 600 °C. Figure 6a,c show the stable and reversible response transients under the cross-input conditions of air and HCHO gas with various concentrations ranging from 10 to 500 ppm, operating at 300 °C and 400 °C, respectively, clarifying the stability and reproducibility of the sensor. The sensor exhibits typical p-type sensing behavior, with an increase in electrical resistance towards the reducing HCHO gasses. It is found that the response values (S) acquired under the operating conditions of 300 °C and 400 °C are strongly dependent on gas concentration, as shown by the responses plotted as a function of gas concentration on a log–log scale (Figure 6b,d). Particularly, the responses show sharply increased values towards 500 ppm HCHO, with values of 3.2 at 300 °C and 3.9 at 400 °C. Such a rapid increase in response values indicates that the concentration required for the HCHO gas to sufficiently adsorb and react on the sensor surface is at least 500 ppm. On the other hand, the response measured at the lower operating temperature of 200 °C exhibits a very low value of about 1.8 towards 500 ppm HCHO. At higher operating temperatures of 500 °C and 600 °C, the responses are close to 1, indicating little to no sensitivity.

**Figure 7:**
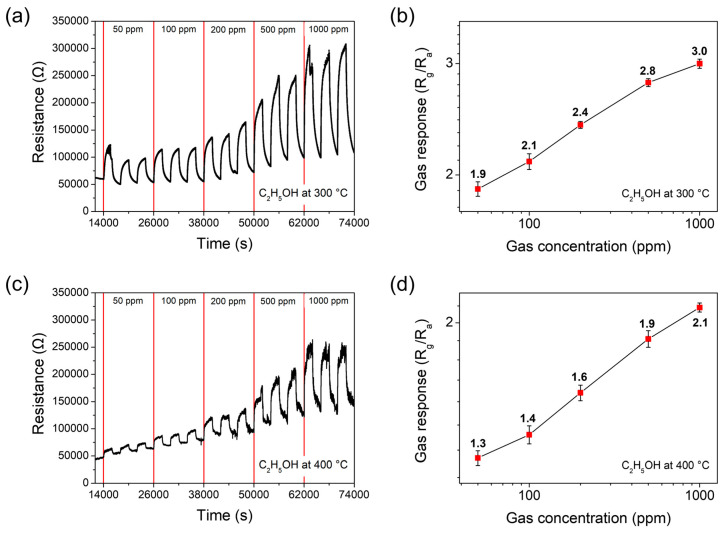
Response transients of the Co_9_S_8_ film sensor which are acquired at various concentrations of 50 ppm, 100 ppm, 200 ppm, 500 ppm, and 1000 ppm at operating temperatures of (**a**) 300 °C and (**c**) 400 °C towards ethanol (C_2_H_5_OH) gas. The gas response values of the sensor are shown as a function of gas concentration on a log–log scale, measured at (**b**) 300 °C and (**d**) 400 °C.

Meanwhile, the sensor responded to ethanol (C_2_H_5_OH) gas. Figure 7a,c exhibit the response transients under the cross-input conditions of C_2_H_5_OH and air with various concentrations ranging from 50 to 1000 ppm, operating at 300 °C and 400 °C, respectively, with three repeated measurements of the curves. Also, p-type sensing behavior is observed for the reducing C_2_H_5_OH gasses. Interestingly, unlike the response to HCHO, it is found that the response to C_2_H_5_OH depends roughly linearly on the gas concentration when plotted on a log–log scale (Figure 7b,d). At the other operating temperatures of 200 °C, 500 °C, and 600 °C, no significant response values were obtained.

**Figure 8:**
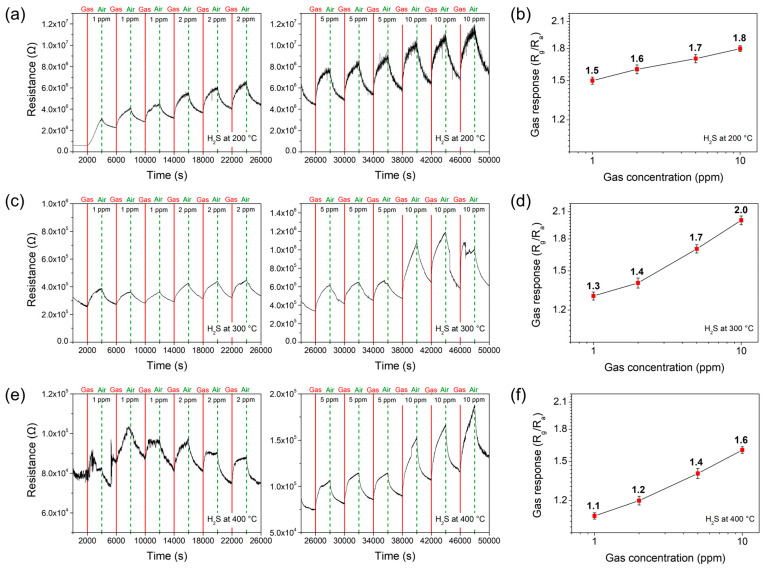
The response transients of the Co_9_S_8_ film sensor which are acquired at various concentrations of 1 ppm, 2 ppm, 5 ppm, and 10 ppm at operating temperatures of (**a**) 200 °C, (**c**) 300 °C, and (**e**) 400 °C towards hydrogen sulfide (H_2_S) gas. The gas response values of the sensor are shown as a function of gas concentration on a log–log scale, measured at (**b**) 200 °C, (**d**) 300 °C, and (**f**) 400 °C.

Towards the reducing hydrogen sulfide (H_2_S) gas, the sensor response reveals a gradual increase with an increase in gas concentration from 1 to 10 ppm, similarly to operating temperatures of 200 °C, 300 °C, and 400 °C (Figure 8).

The sensor responded to hydrogen (H_2_) gas, displaying reversible response transients with gas-concentration dependence at various operating temperatures ranging from 300 °C to 600 °C (Figure S4). However, the response values are very low, and at concentrations below 100 ppm, the response transients are somewhat unstable. Additionally, we found that the sensor shows no response towards 50–1000 ppm acetone (CH_3_COCH_3_) and 1–10 ppm nitrogen dioxide (NO_2_) at operating temperatures ranging from 200 °C to 600 °C.

The magnitude of the gas response (S) was defined as the ratio (R_a_/R_g_) of the resistance in air (R_a_) to that in the sample gas (R_g_). However, as shown in the gas-sensing results, S approaches 1 at the lowest concentrations (S = 1 indicates no gas response). Nevertheless, response curves are weakly detected with S values close to 1 at these concentrations. Based on these results, although we have not explicitly determined the limit of detection (LOD) values, it can be inferred that the lowest detectable concentrations of the target gasses correspond roughly to the LOD for each target gas.

The Co_9_S_8_ film sensor is considered to follow the typical mechanism of a semiconductor-type gas sensor [26,27,65,66]. In ambient air, the O_2_ molecules are adsorbed in the form of O^2−^ or O^−^ on the surface and grain boundaries of the p-type semiconductor Co_9_S_8_, forming hole accumulation layers. When the sensor is exposed to a reducing gas, the pre-adsorbed oxygen ions are removed through a reaction with the reducing gas while donating electrons to the semiconductor. This leads to a decrease in the width of the hole accumulation layers, resulting in an increase in the electrical resistance of the sensor. The detailed reactions of the sensor with reducing gasses such as HCHO, C_2_H_5_OH, H_2_S, and H_2_ are as follows [26,27,66,67]:

(3) $$(HCHO{)}_{gas}+2(O^{-}{)}_{adsorption}\to (CO_{2}{)}_{gas}+(H_{2}O{)}_{gas}+2e^{-}$$

(4) $${\left(C_{2}H_{5}OH\right)}_{gas}+6{\left(O^{-}\right)}_{adsorption}\to 2{\left(CO_{2}\right)}_{gas}+3{\left(H_{2}O\right)}_{gas}+6e^{-}$$

(5) $$2(H_{2}S{)}_{gas}+6(O^{-}{)}_{adsorption}\to 2(H_{2}O{)}_{gas}+2{\left({SO}_{2}\right)}_{gas}+{6e}^{-}$$

(6) $${\left(H_{2}\right)}_{gas}+{\left(O^{-}\right)}_{adsorption}\to {\left(H_{2}O\right)}_{gas}+e^{-}$$

**Figure 9:**
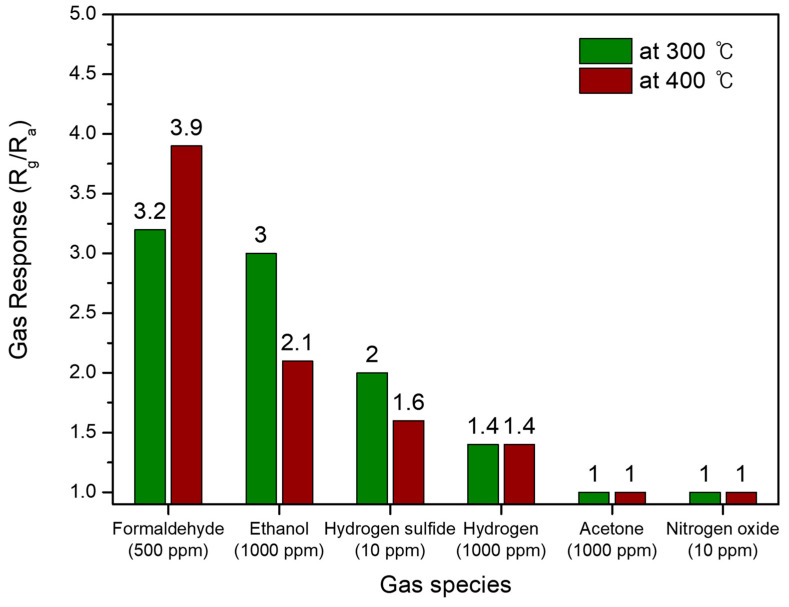
The gas response values for each target gas obtained using the Co_9_S_8_ film sensor.

Given the gas reactions on the sensor surface, the number of released electrons and the adsorption/desorption kinetics can be considered key factors influencing the sensor’s selectivity, although these mechanisms remain unclear. Further detailed studies on selectivity will be necessary in the future. The gas response values for each target gas are shown in contrast in Figure 9. As a result, it is found that the fabricated Co_9_S_8_ film sensor exhibits high responsiveness towards formaldehyde (HCHO), ethanol (C_2_H_5_OH), and hydrogen sulfide (H_2_S). However, the response time (t_90%_) was found to be at a poor level overall for the sensor; specifically, the t_90%_ values were 1837 s and 1773 s for 500 ppm HCHO at operating temperatures of 300 °C and 400 °C; 1007 s and 857 s for 1000 ppm C_2_H_5_OH at 300 °C and 400 °C; and 1591 s and 1500 s for 10 ppm H_2_S at 300 °C and 400 °C.

Actually, the semiconductor-type chemiresistive gas sensor characteristics of Co_9_S_8_, especially pristine Co_9_S_8_, are hardly known. To the best of our knowledge, they have been investigated and reported in only three publications [57,58,59]. In Table 1, the reported performance of Co_9_S_8_ semiconductor-type chemiresistive gas sensors is displayed for comparison. Qiu and Wang reported that Co_9_S_8_ nanotubes responded highly to ethanol and acetone gasses, while exhibiting a relatively low response towards formaldehyde [57]. Their results differed from those of this work due to the formation of SO_4_^2−^ species on the Co_9_S_8_ surface during the hydrothermal process. The group of Chen and Jin reported that Co_9_S_8_@In_2_S_3_ hetero-nanostructures show extremely high responsiveness towards triethylamine compared to single-phased Co_9_S_8_ nanododecahedrons [58]. Additionally, they also found high responsiveness towards triethylamine in MXene-loaded Co_9_S_8_@In_2_S_3_ nanocomposites [59]. However, they showed relatively low responses towards ethanol, acetone, and formaldehyde.

In this regard, although more studies are needed in the future, it is worth noting that Co_9_S_8_ may show a very high response value to certain gasses. The performance of Co_9_S_8_-based sensors can be further improved by structural and electronic modifications such as heterostructure/nanostructure formation and elemental doping, as reported in numerous studies [15,16,17,18,19,20,21,22,23,24,25].

## 4. Conclusions

A pristine polycrystalline Co_9_S_8_ film was fabricated by depositing a Co_3_O_4_ film, followed by sulfidation on Si/SiO_2_ substrates. A sintered and collapsed morphology among the particles, a few micrometers in size, was observed in the Co_9_S_8_ film, with a thickness of 440 nm. The prepared Co_9_S_8_ sensor showed high responsiveness towards HCHO, C_2_H_5_OH, and H_2_S at operating temperatures of 300 °C and 400 °C, with strong concentration dependence: 3.2 at 300 °C and 3.9 at 400 °C for 500 ppm HCHO; 3.0 at 300 °C and 2.1 at 400 °C for 1000 ppm C_2_H_5_OH; and 2.0 at 300 °C and 1.6 at 400 °C for 10 ppm H_2_S. However, the sensor showed very low or no responsiveness towards H_2_, CH_3_COCH_3_, and NO_2_. Further investigation of the intrinsic gas-sensing properties of Co_9_S_8_ and its performance improvement through structural and electronic engineering is required.

## Figures and Tables

**Table 1:** Performance comparison of various Co_9_S_8_ semiconductor-type chemiresistive gas sensors.

Material Form	Gas Species	Gas Concentration (ppm)	Operating Temperature (°C)	Gas Response (R_a_/R_g_)	Ref.
Co_9_S_8_ film (sulfidation from Co_3_O_4_ to Co_9_S_8_)	Formaldehyde	500	300	3.2	This work
			400	3.9	
	Ethanol	1000	300	3.0	
			400	2.1	
	Hydrogen Sulfide	10	300	2.0	
			400	1.6	
	Hydrogen	1000	300	1.4	
			400	1.4	
	Acetone	1000	300	1.0	
			400	1.0	
	Nitrogen oxide	10	300	1.0	
			400	1.0	
Co_9_S_8_ nanotubes with SO_4_^2−^ surface species (hydrothermally synthesized)	Formaldehyde	100	160	1.801	[57]
	Ethanol	100	160	7.326	
	Acetone	100	160	2.065	
	Nitrogen oxide	100	160	1.162	
Co_9_S_8_@In_2_S_3_ hetero-nanostructures (hydrothermally synthesized)	Triethylamine (TEA)	20	300	103.28	[58]
		50	300	307.04	
Co_9_S_8_ nanododecahedrons (hydrothermally synthesized)		20	300	2.638	
		50	300	3.098	
Mxene-loaded Co_9_S_8_@In_2_S_3_ nanocomposites (hydrothermally synthesized)	Triethylamine (TEA)	20	350	44.9	[59]
	Trimethylamine	20	350	2.17	
	Ammonia	20	350	1.63	
	Ethanol	20	350	1.60	
	Ethylamine	20	350	1.38	
	Toluene	20	350	1.37	
	Diethylamine	20	350	1.32	
	Acetone	20	350	1.17	
	Methanol	20	350	1.13	
	Formaldehyde	20	350	1.11	
	Xylene	20	350	1.04	

## Data Availability

The original contributions presented in the study are included in the article and Supplementary Materials, further inquiries can be directed to the corresponding author.

## Supplementary Materials

The following supporting information can be downloaded at: https://www.mdpi.com/article/10.3390/ma17235743/s1, Figure S1: Schematic of the thermal metal-organic deposition (MOD) of Co_3_O_4_ films, followed by sulfidation to prepare the Co_9_S_8_ film. Illustration of the fabricated gas sensor and gas sensor measurement system; Figure S2: FE-SEM images of Co_3_O_4_ films deposited on Si/SiO_2_ substrates by the MOD process using various amounts of cobalt(II) acetate tetrahydrate as the precursor, specifically 10 mg, 20 mg, 30 mg, and 40 mg; Figure S3: The elemental composition of the Co_9_S_8_ film confirmed by EDS; Figure S4: Response transients of the Co_9_S_8_ film sensor which are acquired with the various concentrations of 50 ppm, 100 ppm, 200 ppm, 500 ppm, and 1000 ppm at the operating temperatures of 300 °C, 400 °C, 500 °C, and 600 °C towards hydrogen (H_2_) gas.
