# Supplementary material for: Gas-Sensing Properties of Co9S8 Films Toward Formaldehyde, Ethanol, and Hydrogen Sulfide
Source: Materials (Basel). 2024 Nov 24;17(23):5743. doi: 10.3390/ma17235743 (PMC11642285; doi:10.3390/ma17235743)
Supplement: Supplementary file 1 [file materials-17-05743-s001.zip › materials-3286768-supplementary.pdf]

Supplementary Material

Gas-sensing properties of Co_9S_8 films toward formaldehyde, ethanol, and hydrogen sulfide

Myeong Gyu Kim[1], Yun-Hyuk Choi[1,2,3,4]*

[1]Department of Advanced Materials and Chemical Engineering, Graduate School, Daegu Catholic University, Gyeongsan, Gyeongbuk 38430, Republic of Korea

[2]Department of Energy Materials, Daegu Catholic University, Gyeongsan, Gyeongbuk 38430, Republic of Korea

[3]Department of Battery Engineering, Daegu Catholic University, Gyeongsan, Gyeongbuk 38430, Republic of Korea

[4]Department of Materials Science and Engineering, Daegu Catholic University, Gyeongsan, Gyeongbuk 38430, Republic of Korea

*Author to whom correspondence should be addressed; E-mail: yunhyukchoi@cu.ac.kr (Y.-H. Choi)

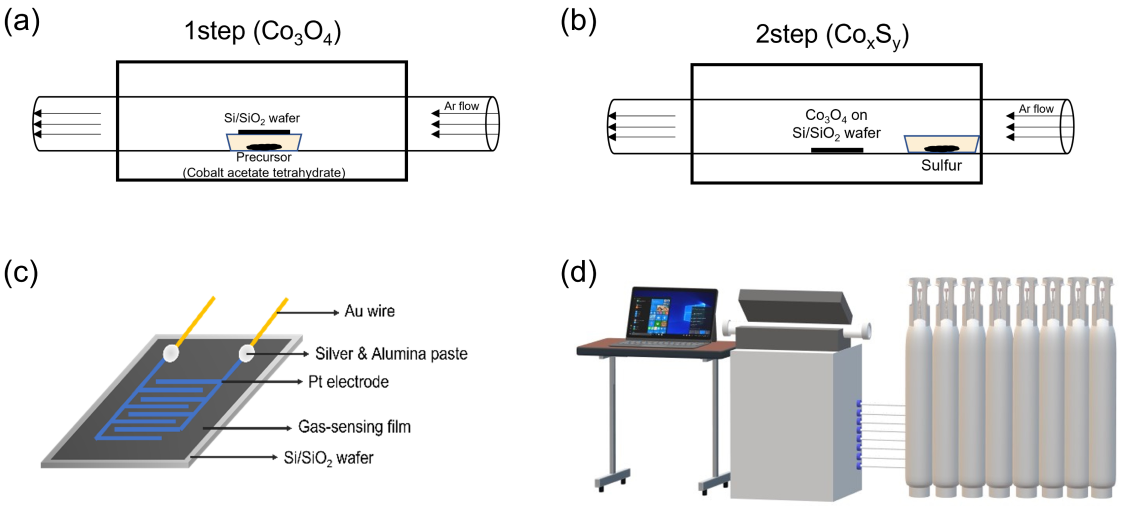

Figure S1. Schematic of (a) the thermal metal-organic deposition (MOD) of Co_3O_4 films, followed by (b) sulfidation to prepare the Co_9S_8 film. (c) Illustration of the fabricated gas sensor and (d) gas sensor measurement system.

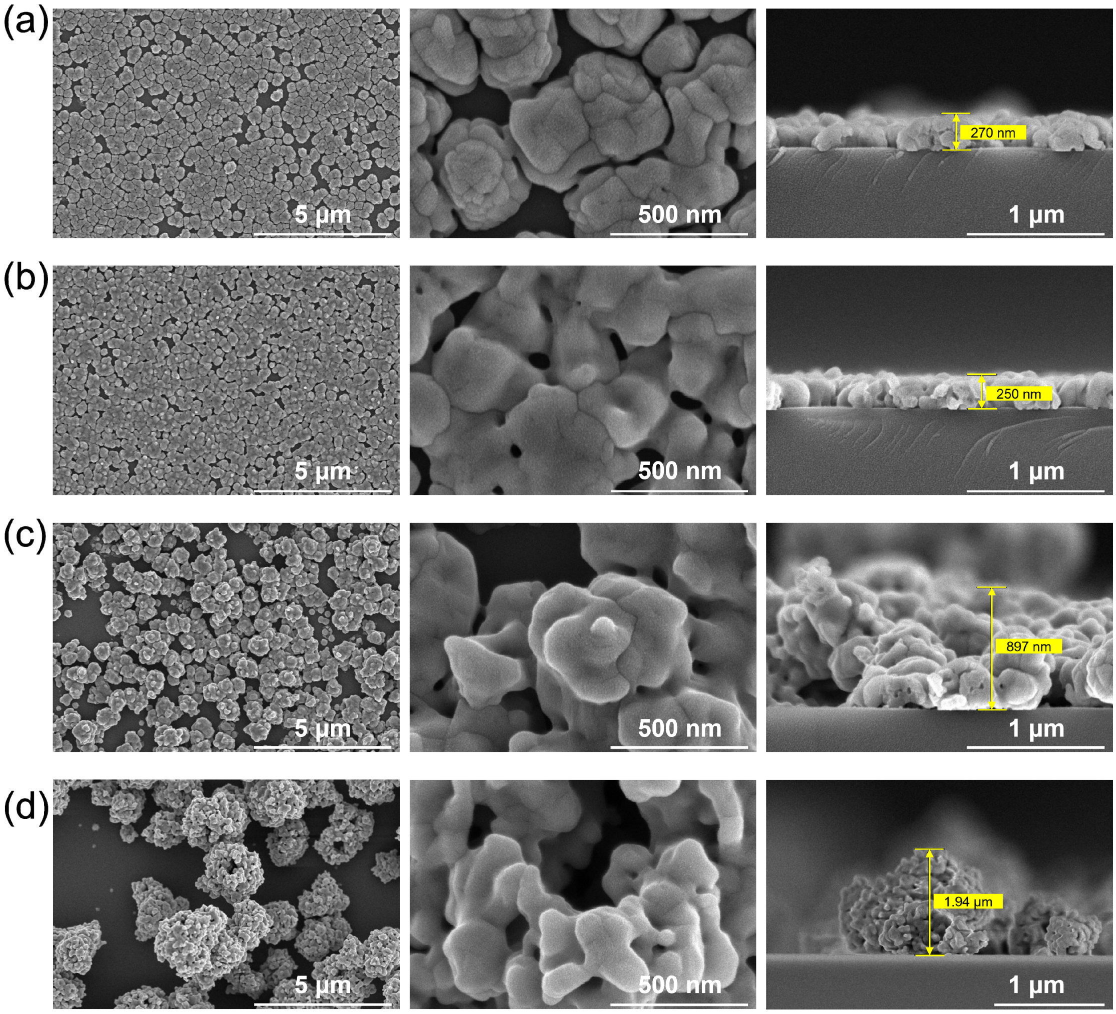

Figure S2. FE-SEM images of Co_3O_4 films deposited on Si/SiO_2 substrates by the MOD process using various amounts of cobalt(II) acetate tetrahydrate as the precursor, specifically (a) 10 mg, (b) 20 mg, (c) 30 mg, and (d) 40 mg. (Left) Top views, (middle) high-magnifications of the left images, and (right) cross-sections.

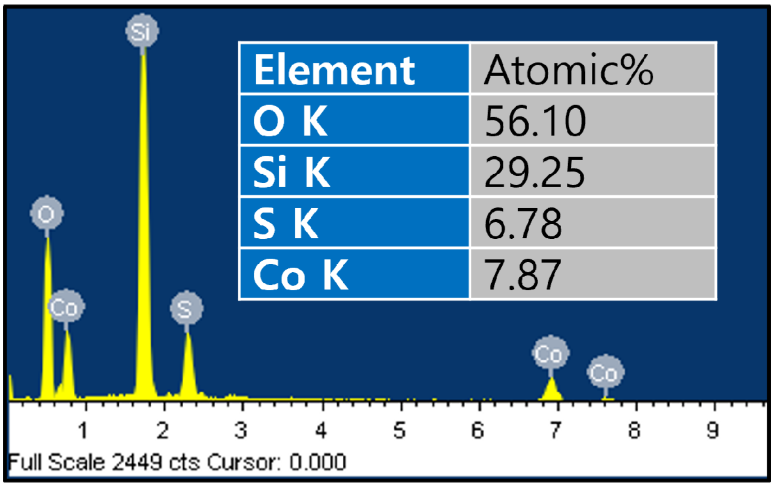

Figure S3. The elemental composition of the Co_9S_8 film confirmed by EDS.

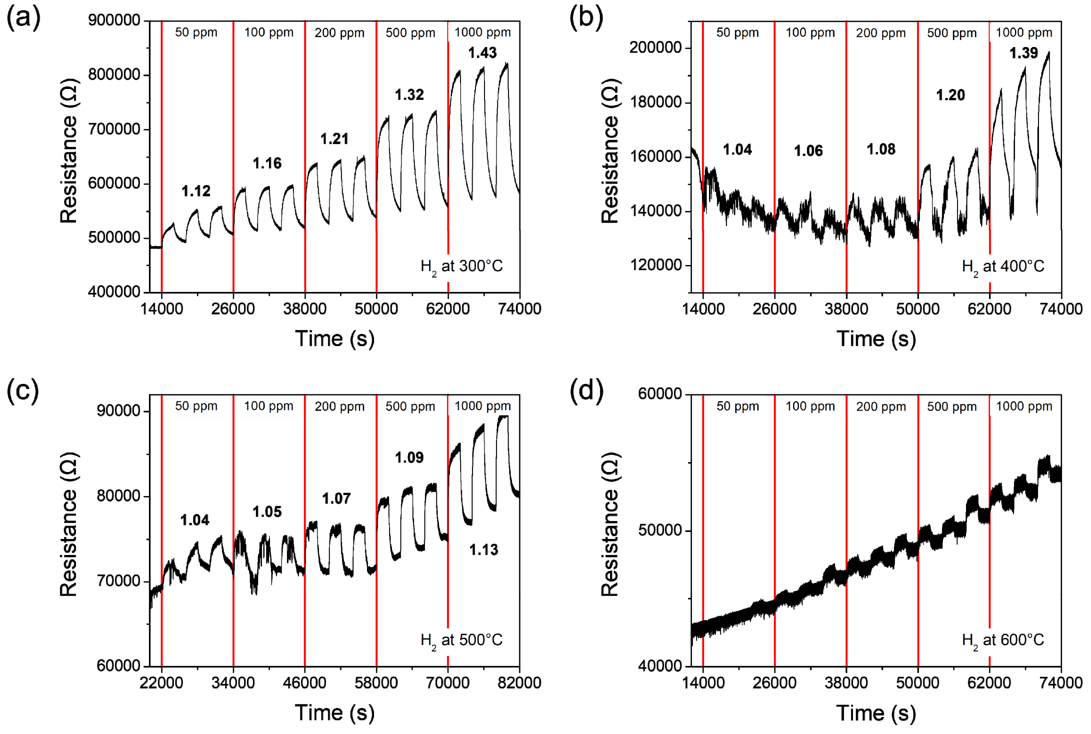

Figure S4. Response transients of the Co_9S_8 film sensor which are acquired with the various concentrations of 50 ppm, 100 ppm, 200 ppm, 500 ppm, and 1000 ppm at the operating temperatures of (a) 300 °C, (b) 400 °C, (c) 500 °C, and (d) 600 °C towards hydrogen (H_2) gas.
